# Comparison of Manual, Semi-Automatic, and Automatic CT-Based Methods for Liver Volume Segmentation

**DOI:** 10.3390/diagnostics16050817

**Published:** 2026-03-09

**Authors:** Berna Dogan, Sadik Bugrahan Simsek, Sefa Sonmez, Merve Nur Ozgen Sonmez, Omur Dasci, Zafer Ozmen

**Affiliations:** 1 Department of Anatomy, Faculty of Medicine, Tokat Gaziosmanpasa University, Tokat 60100, Türkiye; s.bugrahansimsek@gmail.com (S.B.S.); sefa.sonmez@gop.edu.tr (S.S.); mervenur.ozgen@gop.edu.tr (M.N.O.S.); 2 Department of Radiology, Faculty of Medicine, Tokat Gaziosmanpasa University, Tokat 60100, Türkiye; omur.dasci@gop.edu.tr (O.D.); zafer.ozmen@gop.edu.tr (Z.O.)

**Keywords:** liver volume, computed tomography, semi-automatic segmentation, automatic segmentation, deep learning

## Abstract

**Background/Objectives**: To evaluate whether semi-automatic and automatic CT-based liver segmentation methods can provide clinically acceptable volumetric agreement compared with manual segmentation while improving processing efficiency in routine practice. **Methods**: CT images from 86 individuals were retrospectively analyzed. Liver volumes were calculated using manual segmentation, RVX Semi-Automatic, RVX Deep Learning, and TotalSegmentator. Differences among methods were assessed using repeated-measures ANOVA. Agreement with manual segmentation was evaluated using a Bland–Altman analysis, while the Dice Similarity Coefficient (DICE) and Hausdorff Distance (HD) quantified spatial overlap and boundary deviation, respectively. Processing times were recorded. **Results**: Mean liver volumes were 1503.9 ± 356.0 cm^3^ (manual), 1512.6 ± 373.6 cm^3^ (RVX Semi-Automatic), 1549.8 ± 367.9 cm^3^ (RVX Deep Learning), and 1518.3 ± 365.8 cm^3^ (TotalSegmentator). RVX Deep Learning produced significantly higher volumes compared with manual segmentation (*p* < 0.001), whereas RVX Semi-Automatic and TotalSegmentator showed no significant differences (*p* > 0.05). DICE values were 0.911 ± 0.032, 0.946 ± 0.018, and 0.938 ± 0.021 for RVX Semi-Automatic, RVX Deep Learning, and TotalSegmentator, respectively. HD values were highest for the deep learning-based method. Processing times were shortest for RVX Deep Learning and longest for manual segmentation. **Conclusions**: Semi-automatic and automatic liver segmentation methods substantially reduce processing time while maintaining clinically acceptable volumetric agreement. Among the evaluated approaches, TotalSegmentator showed the closest agreement with manual segmentation, supporting its use in routine CT-based liver volumetry. Deep learning-based segmentation, although faster, tended to overestimate volume, potentially limiting its use in applications requiring high volumetric precision.

## 1. Introduction

Accurate and reliable assessment of liver volume has become increasingly important in clinical decision-making, particularly in the context of liver transplantation, major hepatectomy, and complex hepatic surgical procedures [1,2]. Precise volumetric evaluation is essential for estimating functional liver reserve, optimizing surgical planning, and reducing postoperative complications. In addition, liver volume measurement has gained relevance as an objective imaging-based parameter in the assessment of various hepatic conditions [3,4,5].

Computed tomography (CT) is the most commonly used imaging modality for liver volumetry [6]. Although manual segmentation is traditionally used as a clinical reference standard, it requires slice-by-slice delineation, is therefore time-consuming and operator-dependent, and does not represent an absolute ground truth [7,8,9]. Moreover, anatomical complexity and interindividual variability—particularly in the region of the porta hepatis—pose additional challenges for accurate segmentation [5]. To overcome these limitations, various semi-automatic and automatic segmentation methods have been developed, including deep learning-based approaches that aim to reduce processing time and enhance reproducibility. However, despite promising technical performance, these methods have not yet been widely adopted in routine clinical practice, mainly due to limited comparative validation and the lack of standardized evaluation across different segmentation strategies [10,11,12,13,14].

In this study, manual, semi-automatic, deep learning-based automatic, and automatic liver segmentation methods were systematically compared using contrast-enhanced CT images. The primary aim was to determine whether semi-automatic and automatic segmentation approaches can achieve clinically acceptable volumetric agreement comparable to manual segmentation.

## 2. Materials and Methods

This study was conducted in accordance with the principles of the Declaration of Helsinki. Ethical approval was obtained from the Tokat Gaziosmanpasa University Non-Interventional Clinical Research Ethics Committee (No: E-15235480-050.04-564671).

### 2.1. Study Population

In this retrospective study, contrast-enhanced abdominal CT images obtained between January 2025 and May 2025 at Tokat Gaziosmanpasa University Research and Application Hospital were reviewed. Images of healthy individuals who underwent abdominal CT scans for control purposes and had no known disease diagnosis were included. For each participant, anonymized demographic data, including age and sex, were recorded. Based on this list, the Picture Archiving and Communication System (PACS) database was accessed under the supervision of a radiologist. CT images that provided a precise evaluation of liver anatomy were included in the study. Images showing pathological formations in the abdominal region, hepatomegaly, a history of liver disease, or significant imaging artifacts were excluded from the analysis. According to these inclusion and exclusion criteria, abdominal CT images from a total of 86 individuals (41 males, 45 females) were included in the analysis.

### 2.2. CT Imaging Protocol

All abdominal CT images included in this study were obtained from a single institution using a 128-slice multidetector CT scanner (Optima CT660, GE Healthcare, Milwaukee, WI, USA). The imaging parameters were as follows: tube voltage of 120 kV, tube current of 150 mAs, collimation of 64 mm × 0.5 mm, slice thickness of 2.5 mm, reconstruction thickness of 1.25 mm, matrix size of 512 × 512 pixels, and gantry angle of 0°. For all participants, an iodinated contrast agent was administered intravenously at a dose of 1.5 mL/kg. Imaging was performed approximately 60 s after injection, in the portal venous phase, in the craniocaudal direction. Liver assessments were conducted during this phase, when maximum enhancement of the portal and hepatic veins within the parenchyma was achieved.

### 2.3. Segmentation Methods

All segmentation procedures were performed using modules available in the open-source software 3D Slicer (version 5.8.1; https://www.slicer.org/ (accessed on 1 April 2025)). The processing steps for each segmentation method are described below.

#### 2.3.1. Manual Segmentation

Manual segmentation was performed using the Segment Editor module in 3D Slicer. Contrast-enhanced abdominal CT images in Digital Imaging and Communications in Medicine (DICOM) format were imported into the software. Each image was evaluated simultaneously in the axial, coronal, and sagittal planes. A new segment was created using the *Add* option in the *Segment Editor* module, and the liver was manually delineated slice by slice using the *Paint* tool. After completion of the labeling process, a three-dimensional model of the segment was generated using the *Show 3D* option. Volume measurements were automatically calculated through the *Segment Statistics* module.

#### 2.3.2. Semi-Automatic Segmentation with the RVX Liver Module

For this segmentation, the R-Vessel-X (Robust Vascular Network Extraction and Understanding within Hepatic Biomedical Images, RVX) Liver Segmentation extension integrated into 3D Slicer was used [15]. The method was performed using a semi-automatic segmentation approach. First, CT images in DICOM format were imported into the software. Patient data were opened through the *Load DICOM* or *Load Data* modules within the RVX Liver Segmentation extension. Under the *Liver* tab, the *Paint* tool was used to manually label liver and non-liver regions with different colors. After the painting process, the *Initialize* option under the *Grow from Seeds* tab was selected. Any segmentation errors were manually corrected. Finally, liver volume was automatically calculated using the *Segment Statistics* module.

#### 2.3.3. Deep Learning-Based Segmentation with the RVX Liver Module

**Figure 1:**
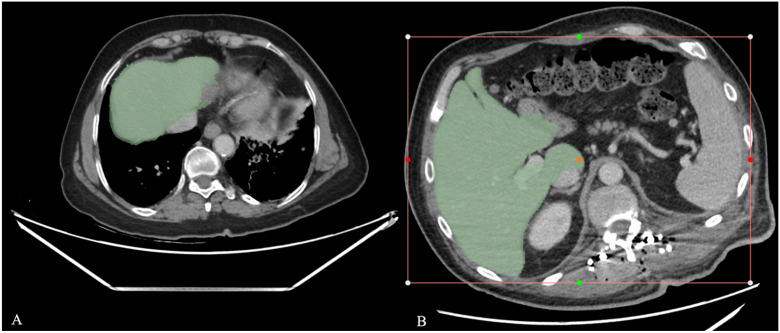
(**A**,**B**) Examples of erroneous segmentations obtained using the RVX Semi-Automatic (**A**) and RVX Deep Learning (**B**) methods. Images were taken from different cases and at different slice levels. Green areas represent the liver masks generated by the segmentation results.

After importing the CT images into 3D Slicer, the RVX Liver Segmentation module was used for liver segmentation. In the *Data* tab, threshold settings were applied as a preprocessing step. Then, under the *Liver* tab, the *Segment CT* command within the module was executed. To define segmentation boundaries, the *Volume Rendering Region of Interest (ROI)* option in the ROI sub-tab was selected and visualized using the *Toggle ROI Visibility* command. The liver region was manually adjusted using visual bounding boxes to include only the target organ. The segmentation process was finalized using the *Apply* command, and the liver volume was automatically calculated through the *Quantification* and *Segment Statistics* modules, located under the *Modules* menu. In both the RVX Semi-Automatic and RVX Deep Learning methods, when segmentation errors occurred (Figure 1), excess regions outside the liver boundaries were removed using the *Erase* tool, and missing parenchymal areas were added with the *Paint* tool. These corrections were limited to distinct errors; minor contour refinements were not performed.

#### 2.3.4. Automatic Segmentation with the TotalSegmentator Module

**Figure 2:**
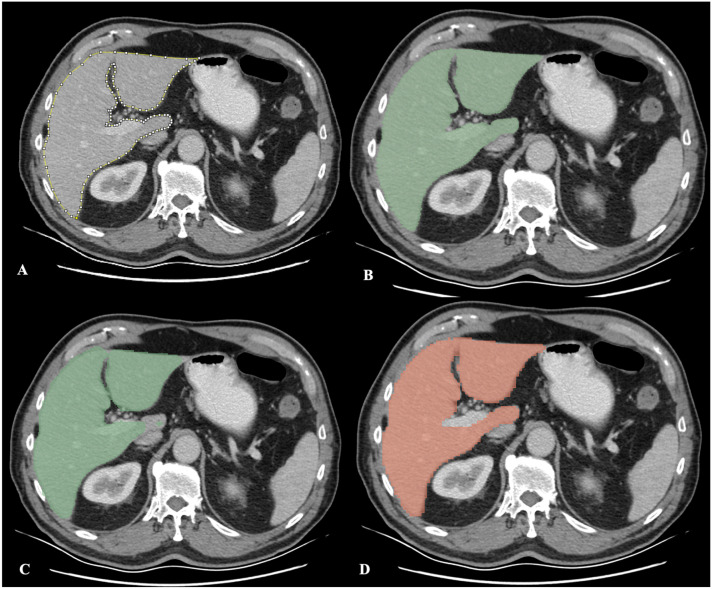
Liver segmentation on the same CT slice using four different methods. (**A**) Manual segmentation performed using the Segment Editor module in 3D Slicer software. (**B**) Semi-automatic segmentation using the “Grow from Seeds” function within the RVX Liver Segmentation. (**C**) Segmentation using the RVX Liver Segmentation by deep learning. (**D**) Automatic segmentation performed using the TotalSegmentator module. Each method illustrates the delineation of liver boundaries on the same axial CT slice for visual comparison.

After importing the CT images into 3D Slicer, the *TotalSegmentator* module [16] was selected. Segmentation was initiated using the *Fast* option, and the *Apply* command was executed to automatically separate the relevant anatomical structures, including the liver, into their respective segments. Three-dimensional visualization of the segmentation was provided using the *Show 3D* option. Once the process was completed, liver volume was automatically calculated using the *Quantification* and *Segment Statistics* modules. Examples of the four segmentation methods applied to the same axial CT slice are presented comparatively in Figure 2.

### 2.4. Segmentation Agreement Analysis

After completing all segmentation procedures, the liver masks obtained from each method were exported in nii.gz format. The Dice Similarity Coefficient (DICE) and Hausdorff Distance (HD) values were calculated using the *SliverRTextension* available in 3D Slicer. Within the software, the *Segment Comparison* module under the *Radiotherapy* section was accessed, and manual segmentation was defined as the reference segment. The comparison segments generated by each method (RVX Semi-Automatic, RVX Deep Learning, and TotalSegmentator) were uploaded individually. The options *Compute Hausdorff Distance* and *Compute DICE Metrics* were enabled, and the measurements were computed automatically. The resulting DICE and HD values were used to quantitatively evaluate the spatial agreement of each method with manual segmentation.

### 2.5. Statistical Analysis

Statistical analyses were performed using IBM SPSS Statistics software (version 28.0; IBM Corp., Armonk, NY, USA). Intra- and inter-observer reliability for the manual and semi-automatic segmentation methods was assessed using Intraclass Correlation Coefficients (ICCs) based on a two-way random-effects model with absolute agreement. For inter-observer analysis, segmentations were independently performed by two observers experienced in abdominal imaging and blinded to each other’s results. For intra-observer analysis, one observer repeated the segmentation after a minimum interval of two weeks. ICC values were interpreted as follows: <0.5 (poor), 0.5–0.75 (moderate), 0.75–0.9 (good), and >0.9 (excellent reliability). Data normality was assessed using the Kolmogorov–Smirnov test. As the data were normally distributed, differences in liver volumes among the four segmentation methods were evaluated using repeated-measures ANOVA with Bonferroni correction for multiple comparisons. Descriptive statistics were presented as mean ± standard deviation (SD). Each segmentation method was compared with manual segmentation. Absolute volume error was calculated as Vmanual − Vautomatic, and relative volume error as (Vmanual − Vautomatic)/Vmanual × 100 [17]. Absolute and relative volume differences were illustrated using boxplots showing the median and interquartile range. A *p*-value < 0.05 was considered statistically significant. Bland–Altman analysis was performed to assess volumetric agreement between manual segmentation and each alternative method by estimating the mean bias and 95% limits of agreement (bias ± 1.96 SD).

## 3. Results

The CT images of 41 male participants (mean age, 60.02 ± 17.32 years) and 45 female participants (mean age, 54.44 ± 18.29 years) were included in this study. There was no significant difference in mean age between the sexes (*p* = 0.151).

Two independent observers performed the manual and RVX Semi-Automatic segmentation procedures. Intra-observer reliability was assessed by repeating the segmentation of a randomly selected subset of 10 cases at two different time points, yielding ICC values of 0.997 and 0.996 for the two observers, respectively. Inter-observer reliability was evaluated by comparing the segmentations of the same subset performed by both observers. The results indicated excellent agreement for the manual method (ICC = 0.991) and good agreement for the semi-automatic method (ICC = 0.889). For subsequent analyses, the mean of the two observers’ volume measurements was used for these methods.

Repeated measures ANOVA was conducted to compare liver volumes obtained from the four segmentation methods. According to Mauchly’s Test of Sphericity, the assumption of equal variances was not met (*p* < 0.001). Therefore, the Greenhouse–Geisser correction was applied for interpretation. The analysis revealed that the segmentation method had a significant effect on liver volume measurements (F (2.348, 199.618) = 18.501, *p* < 0.001, η^2^ = 0.179).

The mean liver volumes (cm^3^), standard errors, and 95% confidence intervals (Lower–Upper Bound) for the different segmentation methods are presented in Table 1. Similar mean volume values were obtained among the methods, with the highest average liver volume observed using the RVX Deep Learning method.

The pairwise comparison of liver volumes obtained using the four segmentation methods is presented in Table 2. After applying the Bonferroni correction, liver volume measured by the RVX Deep Learning method was found to be significantly higher than that obtained with the manual, RVX Semi-Automatic, and TotalSegmentator methods (*p* < 0.05). No significant differences were observed among the other method pairs (*p* > 0.05).

**Figure 3:**
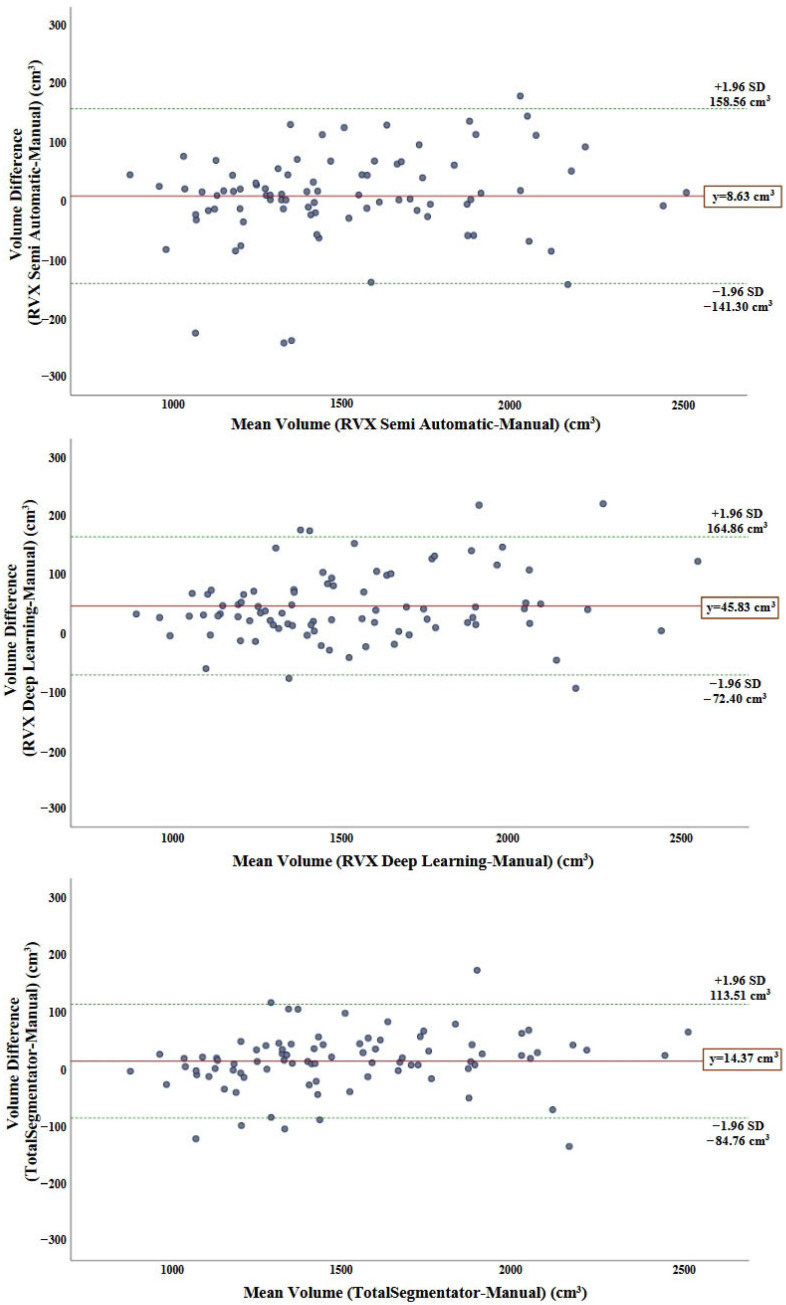
Bland–Altman plots showing the volume differences (New Method—Manual Method) between manual liver segmentation and each of the three methods —TotalSegmentator, RVX Deep Learning, and RVX Semi-Automatic—plotted against their mean volume. The solid line represents the mean bias, and the dashed lines indicate the 95% limits of agreement (±1.96 SD).

The volumetric agreement between manual segmentation (reference method) and the RVX Semi-Automatic, RVX Deep Learning, and TotalSegmentator methods was evaluated using Bland–Altman analyses, where the differences were expressed as New Method—Manual Method. The mean difference for the RVX Semi-Automatic method was 8.63 cm^3^ (95% CI: −141.30 to 158.56 cm^3^). For the RVX Deep Learning method, the mean difference was 45.83 cm^3^ (95% CI: −72.40 to 164.86 cm^3^), indicating a systematic tendency to overestimate liver volume compared with manual segmentation. The TotalSegmentator method showed a mean difference of 14.37 cm^3^ (95% CI: −84.76 to 113.51 cm^3^). Measurements for all methods were mainly within the 95% limits of agreement; however, the lowest volumetric bias was observed in the RVX Semi-Automatic method. These findings suggest that the RVX Deep Learning method tends to yield higher liver volume estimates, whereas the TotalSegmentator and Semi-Automatic methods produce results closer to manual measurements (Figure 3).

**Figure 4:**
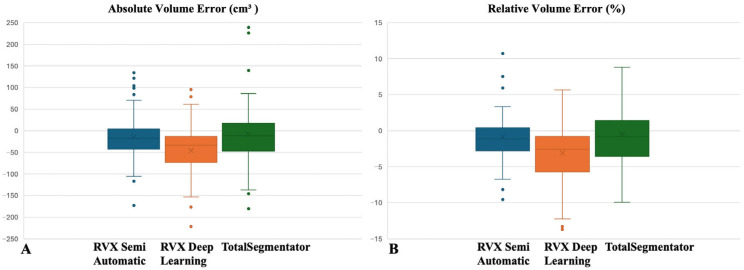
Comparison of absolute (**A**) and relative (**B**) liver volume differences between manual segmentation and the three other segmentation methods using boxplots. The central line represents the median, and the boxes indicate the interquartile range (25th−75th percentiles).

Boxplots illustrating the absolute (Figure 4A) and relative (Figure 4B) liver volume differences between manual segmentation and the other three methods are presented (Figure 4).

The DICE values representing spatial overlap between each segmentation method and manual measurements were 0.911 ± 0.032 for the RVX Semi-Automatic method, 0.946 ± 0.018 for the RVX Deep Learning method, and 0.938 ± 0.021 for the TotalSegmentator method. In addition, the HD values, which evaluate boundary deviation, were 22.74 ± 6.24 mm, 40.26 ± 10.11 mm, and 31.33 ± 9.78 mm, respectively.

When the average segmentation times were evaluated across all methods, the shortest algorithm execution time was observed for the RVX Deep Learning method (0.64 ± 0.09 min). This was followed by TotalSegmentator (2.04 ± 0.47 min), RVX Semi-Automatic (10.42 ± 1.72 min), and manual segmentation (40.42 ± 5.86 min). In segmentations performed using the RVX Deep Learning method, over-segmentation of adjacent anatomical structures was identified in 8 of 86 cases (9.3%). These instances were manually corrected. In the cases requiring correction, the additional processing time was calculated as 5.2 ± 1.6 min. This time was not included in the automatic algorithm execution time. No manual correction was required in the remaining cases.

## 4. Discussion

This study compared manual, semi-automatic, and automatic liver segmentation methods for volume measurement with respect to volumetric agreement, processing time, reproducibility, and potential clinical applicability. Among the evaluated approaches, the RVX semi-automatic method demonstrated the closest agreement with manual segmentation, resulting in minimal volumetric deviation. TotalSegmentator yielded comparable liver volume measurements while substantially reducing processing time, supporting its potential use in routine clinical workflows. Although the RVX Deep Learning method provided the fastest segmentation, it consistently produced higher liver volume estimates than manual measurements, likely reflecting broader boundary delineation by the algorithm.

Several studies have demonstrated that automatic and semi-automatic segmentation methods provide advantages in both agreement and processing efficiency [18,19]. In benchmark datasets, semi-automatic segmentation approaches incorporating user interaction have shown lower variability and fewer outlier errors than automatic systems [20]. High agreement between automatic and manual liver volumetry has been reported in living donor liver transplantation (ICC = 0.994) [21], and a volumetric overlap error of 4.4% for semi-automatic segmentation has been considered acceptable within previously reported ranges [22]. These findings are consistent with the present results and support the reliability of semi-automatic segmentation as an alternative to manual volumetry.

Artificial intelligence-based segmentation algorithms have rapidly evolved, enabling fast and largely user-independent workflows; however, deep learning-based systems may exhibit limited generalizability due to overfitting and dataset variability, underscoring the need for broader validation across diverse imaging conditions [17,23,24,25]. In the present study, although the RVX Deep Learning method achieved the shortest processing time, it consistently produced higher liver volume estimates than manual segmentation, a tendency also reported for deep learning models trained on restricted datasets [17,23]. Algorithm performance is known to vary with anatomical complexity and contrast characteristics; for example, vascular segmentation studies have reported DICE values ranging from 0.75 to 0.83 [26,27]. Although the RVX Deep Learning model achieved a higher overlap coefficient (DICE = 0.94) for liver parenchyma in our cohort, this finding appears to reflect boundary overestimation rather than superior segmentation agreement. The corresponding increase in Hausdorff Distance values further supports the tendency toward boundary extension, leading to higher volumetric estimates.

Fully automatic tools such as TotalSegmentator provide reproducible volumetric measurements without user intervention [28]. In the present study, liver volume estimates obtained using TotalSegmentator did not differ significantly from manual measurements, while offering substantially shorter processing times and minimal operator dependency, supporting its suitability for routine clinical workflows. Automatic segmentation also facilitates surgical planning, treatment monitoring, and longitudinal assessment, providing greater precision than visual inspection or linear measurements in detecting conditions such as organomegaly [5,29].

Reported limits of agreement between CT-based semi-automatic and manual liver segmentation methods vary widely in the literature, ranging from −230 to 327 mL [30], −211 to 278 mL [21], and −503 to 509 mL [31]. Karlo et al. reported limits of −190 to 178 mL in liver resection specimens [32], while Nakayama et al. noted that automatic segmentation may produce larger volumetric errors in damaged livers [30]. Conversely, a recent study reported a mean volume difference of 3 mL with limits of agreement of −117 to 124 mL between semi-automatic and manual segmentation [22]. In the present study, mean differences were approximately 9 cm^3^ for RVX Semi-Automatic (−141 to 159 cm^3^), 14 cm^3^ for TotalSegmentator (−85 to 113 cm^3^), and 46 cm^3^ for RVX Deep Learning (−72 to 164 cm^3^), all within or narrower than previously reported ranges. Among these methods, RVX Semi-Automatic showed the slightest deviation from manual segmentation, whereas TotalSegmentator demonstrated the narrowest limits of agreement and a clear advantage in processing time.

Previous studies have reported substantial variability in liver segmentation time, influenced by factors such as software, hardware, and user experience [2,5,22,31]. In the present study, the RVX Semi-Automatic method reduced measurement time to approximately one-fourth of that required for manual segmentation. At the same time, TotalSegmentator achieved comparable volumetric agreement with an average processing time of about two minutes. Although the RVX Deep Learning method produced results in the shortest time, its volumetric discrepancies relative to manual segmentation warrant cautious interpretation.

This study has several limitations. Although manual segmentation was used as a clinical reference standard, it remains observer-dependent and does not represent an absolute gold standard, as no external ground truth (such as surgical specimen measurements or phantom validation) was available. Therefore, the present study evaluates inter-method agreement rather than absolute volumetric agreement. The single-center design and the use of CT images acquired with uniform acquisition parameters limited the assessment of scanner- or protocol-related effects. In addition, although major vessels were excluded during segmentation, the influence of intrahepatic vessels on volumetric measurements could not be fully controlled, potentially leading to minor variability in measurements. Liver segmentation was intentionally performed using the total liver volume, without subdivision by Couinaud classification or stratification by sex, to maintain a focused comparison of volumetric agreement and processing efficiency across segmentation techniques.

## 5. Conclusions

In conclusion, this study demonstrates that liver segmentation methods differ in their balance between volumetric agreement and processing efficiency. Semi-automatic segmentation achieved the closest agreement with manual measurements, whereas automatic segmentation approaches—particularly TotalSegmentator—provided comparable agreement with substantial gains in processing time. Deep learning-based segmentation, while fastest, tended to overestimate liver volume, underscoring the importance of careful method selection in clinical practice.

## Figures and Tables

**Table 1:** Liver volume measurements obtained using different segmentation methods.

Estimates				
Measure:	Mean (cm^3^)	Std. Error	95% Confidence Interval	
			Lower Bound	Upper Bound
Manual	1503.9	38.382	1427.6	1580.2
RVX Semi-Automatic	1512.6	40.235	1432.5	1592.5
RVX Deep Learning	1549.8	39.629	1470.9	1628.5
TotalSegmentator	1518.3	39.390	1439.9	1596.6

**Table 2:** Pairwise comparisons of liver volume measurement differences among different segmentation methods.

Pairwise Comparisons						
Measure:		Mean Difference (I–J)	Std. Error	Sig. ^b^	95% Confidence Interval for Difference ^b^	
					Lower Bound	Upper Bound
Manual						
	RVX Semi-Automatic	8.63	8.247	1.000	−30.916	13.644
	RVX Deep Learning	45.83 *	6.505	<0.001	−63.402	−28.258
	TotalSegmentator	14.37	5.454	0.060	−29.106	0.363
RVX Semi-Automatic						
	RVX Deep Learning	37.19 *	7.654	<0.001	−57.872	−16.516
	TotalSegmentator	5.73	6.210	1.000	−22.511	11.041
RVX Deep Learning						
	TotalSegmentator	−31.45 *	4.639	<0.001	18.927	43.991

Based on estimated marginal means. *. The mean difference is significant at the 0.05 level. ^b^. Adjustment for multiple comparisons: Bonferroni.

## Data Availability

The data that support the findings of this study are not openly available due to ethical restrictions and are available from the corresponding author upon reasonable request.

## Abbreviations

The following abbreviations are used in this manuscript:

CT	Computed tomography
ICC	Intraclass correlation coefficient
ANOVA	Analysis of variance
DICE	Dice similarity coefficient
HD	Hausdorff distance
SD	Standard deviation
ROI	Region of interest
RVX	Robust vascular network extraction and understanding within hepatic biomedical images
DICOM	Digital imaging and communications in medicine
PACS	Picture archiving and communication system
